# Family dyads, emotional labor, and holding environments in the simulated encounter: co-constructive patient simulation as a reflective tool in child and adolescent psychiatry training

**DOI:** 10.1186/s13034-023-00663-2

**Published:** 2023-10-04

**Authors:** Isaiah Thomas, Laelia Benoit, Robbert Duvivier, Marco Antonio de Carvalho Filho, Andrés Martin

**Affiliations:** 1 Yale School of Medicine, New Haven, CT USA; 2 University of Newcastle, Newcastle, Australia; 3 University Medical Center Groningen, Groningen, The Netherlands

**Keywords:** Patient simulation, Co-constructive patient simulation, Dyadic care, Co-constructivism, Holding environments, Emotional labor, Child and adolescent psyhciatry training

## Abstract

**Background:**

Patient simulation has been used in medical education to provide a safe and supportive learning environment for learners to practice clinical and interpersonal skills. However, simulation involving pediatric populations, particularly in child and adolescent psychiatry, is rare and generally does not reflect the child-caregiver dyad or the longitudinal aspects of this care, nor does it provide learners with an opportunity to engage with and reflect on these dynamics.

**Methods:**

We organized as an educational opportunity a series of seven observed patient simulation sessions with a cohort of a dozen child and adolescent psychiatrists (eight fellows approaching graduation and four senior educators). In these sessions, we utilized the *co-constructive patient simulation* model to create the simulation cases. We included the use of at least two patient actors in most sessions, and two of the case narratives were longitudinally followed across multiple simulation sessions. We approached the data collected during the simulations and their respective debriefings by using thematic analysis informed by a symbolic interactionist approach.

**Results:**

Based on data from the debriefing sessions and longitudinal narratives, we identified four overarching themes: (1) Reflecting on dyadic challenges: role reversal and individuation; (2) Centering the child, allying with the parent, and treating the family system; (3) Ambivalence in and about the parent-child dyad; and (4) Longitudinal narratives and ambivalence over time.

**Conclusion:**

The emotional experience of the simulations, for interviewers and observers alike, provided an opportunity to reflect on personal and professional experiences and triggered meaningful insights and connections between participants. These simulated cases called for *emotional labor*, particularly in the form of creating *holding environments*; in this way, the simulated encounters and the debriefing sessions became *dialogic* experiences, in which the patient and provider, parent and child, and learner and instructor could co-construct meaning and foster professional development as reflective practitioners.

Patient simulation with professional actors or other individuals trained to portray medical encounters (simulated patients) has become increasingly common in pediatric healthcare. For example, undergraduate and graduate medical education offerings routinely include simulations involving delivering difficult news to parents, discussing end-of-life decisions, and navigating concerns of child abuse [1–3]. Simulation facilitates learning patient-centered care by offering less complex clinical situations with a decreased cognitive load that allows learners to focus on communication; a safe environment to experiment and try new approaches without fear of harm to the patient; self-reflection through feedback from supervisors and peers; and development of learners’ sense of self-efficacy [4].

However, two dimensions of pediatric healthcare, particularly in child and adolescent psychiatry (CAP), have been less emphasized in clinical simulation: pediatric patients’ dependence on one or more adult caregivers, and their dynamic developmental trajectories. Buka et al. outlined a number of core principles of early childhood mental health, including: [1] child emotional and behavioral health depends upon a healthy family environment; [2] healthy family environments emerge from healthy early relationships; and [3] parental emotional and behavioral health is essential for a healthy family environment [5]. Similarly, simulating the longitudinal dimension of care in pediatrics and child psychiatry is essential to understanding how patients’ relationships and trajectories change over time.

The Co-constructive Patient Simulation (CCPS) format generates meaningful reflection in medical education by incorporating and integrating multiple points of view [6]. In this model, learners formulate simulated cases together with professional actors using clinical situations that they had found challenging in the past. Extended debriefing sessions are implemented for discussing the emotions triggered by the professional and personal dilemmas presented in the simulation cases. CCPS facilitators emphasize exploring several dimensions of each case and making learning opportunities as explicit as possible for learners. Rather than specific diagnostic or treatment concerns, the focus is exploring and reflecting upon the emotional and interpersonal work that comes with providing care in challenging clinical scenarios [7]. In this way, the model aims to create a simulation space “that offers the trainee the possibility of addressing their own needs” [6].

The *co-constructive* dimension of the model incorporates two theoretical approaches: *co-constructivism*, “the collaborative learning process of co-creating, negotiating, and maintaining meaning through self-reflection and dialogue in a classroom,” and *narrative co-construction*, “the physician’s task of close listening to a patient to coauthor their illness narrative and diagnosis to both center patient agency and remediate preexisting asymmetries of power and expertise” [8]. Given this, the CCPS model seems apt to simulate and reflect upon the demands of dyadic care. Additionally, this co-constructive orientation challenges the hierarchy between learner and expert in the context of medical education; such a mindset lends itself to creating a simulation and reflection space that allows underaged simulated patients to be active participants in all aspects of the process.

We posit that high-fidelity human simulation in CAP could benefit from: (1) two patient actors to simulate the dyadic aspect of the relationship; and (2) recurring sessions with the same clinical case evolving over time. To address these dimensions, we employed the CCPS model as a reflective tool for CAP trainees following the implementation of two innovations to the model: (1) the inclusion of two simulated patients in the same encounter for nearly all simulation sessions; and (2) the use of evolutive clinical cases that spanned 2 to 3 simulation sessions, allowing for a more longitudinal view of the patient’s narrative and development.

The inclusion of children and adolescents as simulated patients (SPs) would not only contribute to the physical fidelity of the simulation (the simulated patient physically resembling and behaving as a patient of a similar age) but also allow the opportunity to appreciate and incorporate their communication preferences, points of view, and priorities into the simulation. Adults frequently make assumptions regarding children’s perceptions of their care; in a study of children ages 11 to 14 exploring what makes a good nurse, the participants expressed a desire to be engaged with as people and a view of overly friendly approaches as off-putting or patronizing [9]. Including underage actors would bring a particular perspective to the role that an adult script writer or adult actor could not [7]. By including their voices in the planning and execution of the simulated encounter, child and adolescent actors could strengthen the authenticity of the simulation. Bokken et al. reported that instructors commented that the adolescent actors drew attention to interesting dimensions of communication, such as “learning to deal with 2 people in a consultation (dividing attention),” “dealing with peers professionally (less formally, yet remaining serious),” “setting personal boundaries in a consultation (with a quarrelling couple),” and “asking questions/talking about sexuality” [10]. Along with contributing to better healthcare for young people at large, Plaksin et al’s review found that many adolescent SPs reported direct benefits from participating in simulation [11]. These SPs appreciated playing an important role in the education of health care providers, learning that adults and healthcare providers can make mistakes, gaining a better understanding of their own communication style and skills, and developing more self-confidence. They also reported increased empathy for others experiencing medical or psychiatric illness as well as a greater ability to discern the quality of healthcare providers.

Despite the direct and indirect benefits of high-fidelity simulation involving caring for and communicating with pediatric patients and their families, there are nevertheless ethical considerations around the inclusion of children and adolescents as simulated patients. For example, some adolescent SPs described transient depressive feelings and needing a few minutes to get out of character after completing psychologically challenging cases [11]. In one study of younger SPs (ages 6 to 9), most participants found the experience to be fun, but one SP reported experiencing fear after considering for the first time the possibility that a child their own age could die [12]. Additionally, at a structural level, child SPs are at times subject to long work hours, coercion, and limited agency in their role; to minimize the risk of harm to underage simulated patients, Budd et al. offer a series of guidelines for involving children in simulation, many of which center around ensuring that children have opportunities to provide input and feedback about the design, rehearsal, delivery, and debriefing of simulation sessions [13]. In a similar vein, Khoo et al. emphasize the importance of ensuring underage actors are engaged as active participants in learning, rather than passive ones [14].

We sought to explore the emotional responses, personal reflections, and collective knowledge generated regarding providing psychiatric care to children and adolescents via the modified CCPS model. We conducted a qualitative analysis of the content discussed during the debriefing sessions after each simulation to investigate how these changes to the CCPS format shaped participants’ reflections and reflected particular aspects of providing dyadic and longitudinal mental health care to children, adolescents, and families.

## Methods

### Study participants and ethics approval

A cohort of eight senior fellows approaching their graduation in CAP and four senior CAP educators elected to participate in a formative educational opportunity involving a series of simulation sessions that were developed according to the CCPS model, with the modifications described above. Given the logistical constraints (including legal restrictions at our institution limiting the recruitment of underage actors to ages 14 to 17) and potential emotional impact of taking part in difficult simulation cases, we elected to utilize older adolescent patient actors and focus on adolescent psychiatry cases. Most cases also included adult actors. All actors had experience working in medical scenarios, and had been trained following standard guidelines in the field [15]. We obtained institutional review board approval from the Yale Human Investigations Committee (Protocol # 2,000,026,241). Trainees were encouraged to participate but informed that their participation was neither mandatory nor pertinent to their fellowship performance evaluation. They were aware that sessions would be conducted as part of a research project and that all interviews and debriefing sessions would be audiotaped, transcribed, and deidentified toward a subsequent qualitative study.

### The CCPS model

Per the CCPS model, preparing the “script” for each simulation session began with a selected “writer” (typically one of the CAP fellow participants) collaborating with facilitators and 1 to 2 actors. Together, they developed a patient profile and story based on one or more challenging clinical scenarios the writer has encountered. This group then rehearsed an improvised clinical encounter based on this profile and story. During the actual simulation session, two interviewers were selected among the child psychiatry trainees and participating attending child psychiatrists. They were blinded to the case other than a “door note” that provides a brief description of the patient and the reason for the encounter at the start of the simulation. The first interviewer began the clinical encounter and interviewed the actor(s) for 20 min. After 20 min, the second interviewer stepped into the role and interviewed the actor(s) for 20 min and completed the encounter. We chose to include two interviewers in each session to demonstrate two different interviewing approaches for the same clinical scenario and to maximize participants’ opportunities to play the role of interviewer while providing sufficient time for depth of interviewing. Facilitators and other participants observed the simulated encounter for its entirety. After the simulated encounter was completed, the interviewers, other participants, facilitators, and patient actors engaged in an hour-long debriefing session to reflect on the encounter [7].

### Data collection

From November 2021 to June 2022, we organized 7 patient simulation sessions as an educational opportunity. Six of the sessions took place in person, and one (January 2022) occurred over Zoom videoconferencing. All sessions and debriefings were digitally recorded and transcribed using Rev.com.

### Data analysis

We conducted an inductive thematic analysis of the data from the simulation sessions and their respective debriefings to develop themes and a working model regarding the reflective experience of participants in CCPS sessions [16]. Our thematic analysis was informed by symbolic interactionism, an epistemological framework that connects social structures with individual-level processes to better understand how individuals interact with one another to create symbolic worlds and how these worlds shape individual behaviors [17]. In Blumer’s formulation, symbolic interactionism “sees meanings as social products, as creations that are formed in and through the defining activities of people as they interact” [18]. Non-symbolic interaction takes place when one responds directly to the action of another without interpreting that action; symbolic interaction requires interpretation of the action. Social interaction is a process that *forms* human conduct instead of being merely a means or a setting for the expression of human conduct. As a source of data, group debriefing sessions (in contrast to individual interviews) can reveal shared beliefs, identities, and collective knowledge, which are particularly meaningful when examining underlying social relationships through symbolic interactionism. Training in child and adolescent psychiatry requires navigating networked interactions and reflexivity and thus calls for trainees to learn to observe interactions, make sense of them, and reshape these interactions.

We first independently analyzed and coded the transcribed and anonymized recordings of the debriefing portions of the 7 simulation sessions. We compared data between debriefing sessions to identify recurring themes, integrate new elements, and ensure triangulation and data sufficiency, that is, the point at which additional analysis only supported identified themes and did not provide new themes or insights. Once sufficiency was achieved, we constructed a complete thematic description of the experiences of the participants, organized into overarching domains linked to underlying themes, each illustrated through verbatim quotations from the debriefing sessions.

## Results

Table 1 provides a brief description of each of the cases written by participants for the simulations. In this results section, we present four themes developed via a symbolic interactionist analysis of the debriefing data, as summarized in Table 2 with example quotations: (1) Reflecting on dyadic challenges: role reversal and individuation; (2) Centering the child, allying with the parent, and treating the family system; (3) Ambivalence in and about the parent-child dyad; and (4) Longitudinal narratives and ambivalence over time.

**Table 1:** Summaries of the seven simulation cases written by participants

Case number	Session date	Scenario description	Number of actors	Learning objectives
1 A	April 2021	Aiden, age 16, has been referred by his school social worker for behavioral concerns at school and home in the setting of his parents’ recent divorce and one parent’s gender transition. The clinician is meeting via videoconference with Aiden and his mother.	1 adolescent, 1 adult	1. To balance parent needs/expectations vs. building rapport with the patient 2. To set appropriate boundaries on inappropriate behavior from patients 3. To become familiar with research-based interventions to improve familial attunement, including approaching non-supportive caregivers with curiosity and concern rather than confrontation with corrective action and creating spaces for families to hear and realize the suffering lack of support creates across generations. 4. To explore gender roles/expectations, and cultural norms for parents and families 5. To become comfortable talking about trans health topics and providing psychoeducation to patients about gender/sex/sexuality.
2 A	June 2021	Sonya, age 15, is currently hospitalized for suicidal ideation and self-injury. The clinician is meeting with Sonya and her father via videoconference to discuss her progress and initiate medication. Sonya has not spoken to her father in six months in the setting of her parents’ divorce and strained relationship.	1 adolescent, 1 adult	1. How to reassess the clinical conclusion of a colleague and give a second opinion? 2. How to communicate alternative clinical judgment to children and families while acknowledging respect for our colleagues? 3. To identify *childism*, which is hidden cruelty and prejudices against children in child-rearing. As physicians, we are mostly trained to inquire about physical violence and severe neglect; but this case is about psychological abuse. 4. To inquire about adverse childhood experiences (witnessing physical violence, experiencing verbal violence, bullying, psychological abuse, and emotional neglect) 3. To assess dissociative and somatic symptoms in teenagers (paralysis, stomachache, vomiting, dizziness) due to chronic traumatization. 5. To navigate the teenagers’ attempts to autonomy (i.e., refusing to see a parent) and the legal visitation requirements. Even more when complex post-traumatic symptoms are clear but there is no physical mistreatment. 6. To inquire about teenagers’ coping strategies: addictions (weed, binge eating), self-harm. 7. Address the complaint of the parent who feels rejected by the child and the other parent. 8. Address some parents’ narcissism, lack of empathy, manipulation, and controlling attitudes.
1B	November 2021	A continuation of case 1 A. Aiden, age 16, and his mother return to the clinic after being lost to follow-up. Aiden continues to struggle at school and home in the setting of his mother’s gender transition, along with the introduction of her partner into their home life.	1 adolescent, 2 adults	
2B	January 2022	A continuation of case 2 A. Sonya, age 15, has been discharged from the hospital and returned to living with her mother. She was supposed to begin outpatient therapy and resume contact with her father. However, she has missed all of her appointments and has not contacted her father. The clinician is meeting with her for the first time today, along with her father, via videoconference to discuss their relationship.	1 adolescent, 1 adult	
3	February 2022	Hala, age 21, has been admitted to a medical floor for altered mental status in the setting of a hypoglycemic episode; she has diagnoses of type 1 diabetes, depression, and anxiety. It is day 3 of her hospitalization and she is progressing toward discharge. However, she experienced another hypoglycemic episode the previous night. The medical team consulted the CAP consult service because they are concerned about the surreptitious use of insulin for self-harm. The team searched Hala’s bag and found several insulin pens; they have not yet discussed their concerns with her. They would like the clinician to evaluate Hala for safety.	1 transitional age adult	1. To explore explicit and implicit biases while providing medical treatment to patients with mental health concerns. 2. To provide experiential opportunity in navigating a complex scenario as a consulting physician when a patient’s privacy or rights have been ignored with “good intentions”. 3. To explore the impact of countertransference on patient care.
4	March 2022	Lisa, age 15, comes to the clinic with her mother. Her mother has wanted to engage in “family therapy” but has not brought her daughter to the last five visits. Previous visits have centered on the mother’s feelings about her ex-husband and Lisa’s father. Today, her mother hopes that her daughter will confirm her suspicions of sexual abuse by her father. Her mother would like the clinician to convince Lisa to disclose the abuse or perform an exam to determine if abuse has occurred.	1 adolescent, 1 adult	1. To experientially explore the role of being a child adolescent psychiatrist when the guardian is unknowingly disrupting a therapeutic environment 2. To gain confidence in setting boundaries to a parent concerning technology and your utility as a clinician 3. To explore feelings of countertransference and how they can positively and negatively impact clinical interactions when a parent is unstable. 4. After the scenario, to discuss the dual role of being a physician and a child advocate
5	April 2022	Toby, age 15, is hospitalized for behavioral concerns at school and at home. On the unit, he has not been allowed to participate in groups because of his use of racist language toward peers and staff. The clinician is meeting with Toby and his grandmother because she is demanding that he return to participating in groups. His grandmother is his primary caregiver; her daughter passed away when Toby was very young. Toby’s grandmother is white, and Toby is mixed race; he has recently discovered that his father, who has not been present in his life, is Mexican.	1 adolescent, 1 adult	1. To explore the ability to be empathic when you don’t agree with a patient or parent’s ideology 2. To analyze your reactions in the face of racism in a patient and parent 3. Expand knowledge on how to address these situations in a therapeutic setting
6	May 2022	Brian, age 15, and his mother come to the clinic due to worsening depression symptoms, at the suggestion of a member of their church, in the setting of his father’s recent death. Brian recently joined the Nation of Islam to feel more connected to his father, who was Black. He has decided to change his name to “Divine X.” His mother, who is white and a devout Christian, is distraught about this decision.	1 adolescent, 1 adult	1. Enhance awareness of one’s limitations and strengths in addressing religious beliefs (which may or may not be maladaptive). 2. Increase comfort and confidence in navigating a patient’s religious and or racial themes during a clinical encounter. 3. Discover ways to increase one’s cultural humility. 4. Explore the conceptualization of normal syndromes of distress as distinct from depression and encompassing grief and or demoralization.
1 C	June 2022	A continuation of cases 1 A and 1B. Aiden, age 16, and his mother return to clinic due to difficulties at home and at school. Aiden was recently admitted for suicidal ideation in the setting of breaking up with his girlfriend. His mother is concerned because Aiden has become more oppositional at home and has begun vaping. He has also been getting into fights with classmates at school.	1 adolescent, 1 adult	1. To balance parent needs/expectations vs. building rapport with the patient 2. To set appropriate boundaries on inappropriate behavior from patients 3. To become familiar with research-based interventions to improve familial attunement, including approaching non-supportive caregivers with curiosity and concern rather than confrontation with corrective action and creating spaces for families to hear and realize the suffering lack of support creates across generations. 4. To explore gender roles/expectations, and cultural norms for parents and families 5. To become comfortable talking about trans health topics and providing psychoeducation to patients about gender/sex/sexuality.

**Table 2:** Themes and illustrative quotations

Theme	Illustrative quotation
**Reflecting on dyadic challenges: role reversal and individuation**	“He was kind of parentifying her too because he kept saying really negative things about mom. And she was placed in a situation where she constantly had to be defending mom” (Case 2B). “I also thought that at 15, kids begin to kind of have their own opinions, and they get into arguments with their parents as a way to separate a little bit more. We worry about what’s going to happen when he leaves and moves out in the world, if I’ll be left all alone” (Case 6).
**Centering the child, allying with the parent, and treating the family system**	“There are also some cases with parents with very high income and high IQ who are able to argue really well. You are balancing between ‘Is the parent abusive?’ or ‘Are the arguments legitimate?’ Who should I protect?” (Case 2B). “I think that when mom does try to enter a holding space for [the patient], her hands are somewhat limited because she’s still trying to hold herself” (Case 1 C). “It shifted it from focusing on the logical piece of the story to what’s the emotional meaning behind this need to protect the daughter and ask about it” (Case 4).
**Ambivalence in and about the parent-child dyad**	“But when mom would say that okay what should I change? What should I do? He would say, oh it’s not about you. So there was this ambivalence about what really was going on” (Case 1 C). “[The father] wants to do well. So the intentions are good and the love is really there, but you can traumatize your kids, even if you love them” (Case 2B).
**Longitudinal narratives and ambivalence over time**	“As I was [the interviewer] in the first session, I was actually taken back for a little bit. I was like, oh well wait, these are the same people, what’s going on? For a second, it took a while to realize that this was a continuation. And I will agree with you that I felt like the energy was kind of down and depressing” (Case 1 C). “I remember I was almost crying after the first session but it was much calmer this time” (Case 1 C).

### Reflecting on dyadic challenges: role reversal and individuation

Across multiple debriefing discussions, participants noted challenges particular to the parent-child relationships, namely role reversal and the process of separation and individuation. With the exception of one, all the cases depicted difficulties of this nature, suggesting that this was a common dynamic that participants had encountered during their training. Participants reflected on the expectations parents placed on children in the simulated relationships and how roles could become reversed with the child being expected to take on the responsibilities of an adult and manage the parent’s emotions:

[The mother] would get stuck with her own guilt. And then at one point, she said, “Should I just die?” And then [the child] responded, “Well, why are you going to put that on me?” And there was a cross just being thrown on his back (Case 1 C).

The term *parentification*, or a child being forced to take on the role of an adult in the parent-child relationship (such as a confidante, mediator, or caretaker) came up during multiple debriefing sessions:

The dad was kind of parentifying his daughter because he kept saying really negative things about mom. And she [the daughter] was placed in a situation where she constantly had to be defending mom (Case 2B).

One participant explained how parentification could come in the form of parents asking their children to “help me to help you.” While this may be an understandable response on the part of parents, it could cause the child to “feel responsible for their relationship” (Case 1 A). Along similar lines, a physically or emotionally absent parent who was attempting to re-enter their child’s life can parentify the child by expecting them to “act like an adult” and immediately be ready to rebuild the relationship:

Now I [as the parent] have gotten my act together, I’m better now, I’m not using substances, I’m out of the depression. I’m unfrozen now. And now I’m going to catch up in this relationship (Case 1 A).

Even as the children in the simulated cases were expected to act like adults, the process of individuation and separation during adolescence was a common source of conflict between parents and children. Participants reflected on the shifting nature of dyadic relationships during this period and the emotional experience of parents struggling with their children growing less dependent on their relationship:

At 15, kids begin to have their own opinions, and they get into arguments with their parents as a way to separate. They worry about what’s going to happen when he leaves and moves out in the world, if they will be left all alone (Case 6).

Even when the cases were ostensibly centered on other issues, such as a parent’s gender transition or a son’s exploration of a new faith, participants readily identified the process of individuation as an underlying source of the tension in the dyadic relationship. As participants attempted to bridge the gaps in understanding between the parent and child, they also recognized that creating space in the dyad could be anxiety-provoking and painful for the parent and experienced as threatening their role as a parent and their relationship with their child. Therefore, participants noted the importance of providing a “safe space” to name and process these emotions, for both the parent and the child:

We could explore things around separation, even if it’s very frightening, because we have this safe space [in the clinical encounter] to explore whatever is frightening for both of them (Case 1 C).

In this case, participants noted that separation and individuation not only represented physical or emotional distance, but also the uncertainty of the future and the unknowability of what might become of a parent-child relationship when the child grows up.

### Centering the child, allying with the parent, and treating the family system

Participants described the particular emotional and interpersonal work that comes with addressing the unrealistic expectations a parent may place on a child or helping a parent come to terms with the fact that their child is growing up. Such work called for both empathizing with the parents’ struggles and centering the child’s needs. Participants reflected on the complex and shifting nature of the answer to the question “Who is the patient?” in these simulated encounters. Many participants expressed wanting to treat the whole family unit while still trying to ensure that they were prioritizing the child:

Is [the patient] the parents or is it the kid? Oftentimes, I’ve defaulted to the kid (Case 1 C).

Centering the child and their needs often meant helping the parent and child hear each other, which was easier said than done. This required ensuring that the child had an opportunity to be heard and preventing the parent from taking up too much space in the dialogue:

I’m sure grandma is just fragile and trying to hold it together. But she was too big in the room and [the patient] was way too small. He was the one who was hospitalized and wanted to kill himself (Case 5).

Participants described toggling back and forth between viewing the child and the parent as two individuals and as a single dyadic unit. In order to center the child, they had to consider how the child fit into the parent-child relationship, which in turn required trying to understand the parent’s motivations and needs and how that might shape the dyad. Participants emphasized the importance of empathy for the parent, for the sake of the child:

It shifted it from focusing on the logical piece of the story to what’s the emotional meaning behind this need to protect the daughter (Case 4).

When working with a parent-child dyad, the interviewers had to contend with the reality of the child and the reality of the parent and try to assemble a coherent understanding of these realities that would be meaningful to everyone involved. One case centered on an adolescent struggling in the context of his mother’s gender transition. In this scenario, the mother and the patient viewed the same event (the mother’s transition) as the start of a new life and as the end of a life, respectively. Both realities could be true for the patient and his mother individually but perhaps could not coexist in the same space at the same time. One participant described the complexity of this bereavement and the recognition of the conflicting realities of the transition for the mother and the child:

Maybe just handling the grieving process with [the child] alone so that he can privately name what he lost very explicitly but without bringing that feeling of discomfort to his mother. For them to handle it together in the same room, I think that would be really, really complicated (Case 1 A).

However, participants could still struggle at certain points to center the patient. In a different session utilizing this same family narrative (Case 1B), the script called for a third actor to play the mother’s new female partner. Over the course of the simulated encounter, the dynamics became emotionally charged and chaotic at times, with the SPs at times interrupting and speaking over one another. For many participants, the accounts of the two adults seemed to trump the child’s experience of the situation; participants tended to frame the adolescent male patient as the aggressor in their comments. Yet during the next simulation and debriefing session (Case 2B), which involved similar challenges in a different parent-child relationship and gender constellation, participants generally came to the adolescent female patient’s defense and framed her father as the aggressor. Participants could identify personal histories that could result in prejudging the parent in a clinical scenario:

I come from a place where my dad was not in my life, so I really have a negative connotation towards men in general, and it’s going to be completely real. So it’s almost like guilty until proven innocent for me (Case 2B).

The differing responses to these two scenarios suggested that in spite of one’s best efforts to center the child in the therapeutic relationship, social factors such as gender stereotypes or virtue signaling towards gender minorities could complicate those efforts:

You had these two women, and one a trans woman, who maybe we [the clinicians] see as even more vulnerable or whatever, and we say no, no, no, I can’t push because if I push, maybe I’m a transphobic jerk (Case 2B).

In the previous session featuring the same adolescent patient and his mother (played by the same actors) but without the mother’s partner present, one participant noted the empathy that the interviewer extended to the struggling patient:

When you said to [the child], “When someone transitions, it’s not just them, it’s everybody around them.” That seemed to be like a big moment of opening for him, where he felt seen and allowed him to speak more about his experience (Case 1 A).

When the dynamics were limited to only the patient and the caregiver, participants seemed to empathize with the child more easily. More specifically, the empathy described above did not only seek to identify what the patient was feeling but also created a space for him to experience his emotions, with an understanding that the patient had as much a right to be having a tough time as anyone else. The interviewer’s comment signaled an awareness of the reciprocal nature of the parent-child relationship: In the same way that a significant shift in a child’s life, positive or negative, can be difficult for a parent to process, a child can struggle to adjust when a parent’s life changes radically.

In the debriefing sessions, participants described the work required in the simulated scenarios as simultaneously holding the needs of both the child and the parent. The CCPS model and the use of two patients appeared to allow participants to engage in and reflect upon the particular approaches that dyadic challenges call for. In keeping with a co-constructivist perspective, participants viewed the emotional work of child and adolescent psychiatry as navigating and negotiating two conflicting realities that nevertheless coexist in the same space and time during the clinical encounter.

### Ambivalence in and about the parent-child dyad

Across multiple sessions, participants noted patients expressing *ambivalence*, or conflicting feelings, toward a parent, feeling simultaneously pulled in and pushed away. At times, the ambivalence could cause children and parents to question their relationships with each other, and at other times, it could cause them to question themselves:

Grandma is in such pain about it and feels such hatred towards his father around the death of her beloved daughter. And then [the grandchild] is in the middle of this horrible dilemma: I want the love of my dad. I want somebody that I can identify with, but my grandma, who I love dearly, hates him and thinks he’s evil. Am I evil?” (Case 5).

My gut feeling at that time was acknowledging that mom’s certainty and intensity is coming from a place of intense love and also hurt at what’s happened to her, and that those are the two forces that are driving this certainty, and just to name them (Case 4).

Even as they felt the urge to take sides, participants experienced feelings of ambivalence toward the parents, the patients, and the family dynamics in the simulated cases. They tried to focus more on the aspects of the encounter that were external to themselves as the clinicians, i.e., what the encounter is bringing to me. When faced with challenging patients, they tried to check their baggage at the door and separate themselves from the encounter:

She’s a grandma, she just had her grandson admitted to a psychiatric hospital for the first time. That’s terrible. Go work with that. Try to ignore everything else (Case 5).

One result of how personal the clinical encounter could become was that the “clinical gaze” of any two clinicians could differ significantly; in our study, two participants could focus on different aspects of a parent’s or child’s actions and have very different interpretations of the same actions:

I felt that the father was abusive: the thought of him hitting her sister, his response. I thought either it was sexual assault or physical abuse because of [the child’s] body language, it looked like somebody that was physically abused (Case 2B).

Contrary to a lot of people, I felt a lot of pain for the dad. I did feel anger towards the dad, but I’d say a majority of it was I felt really bad and really sorry, and maybe a little pitiful for him. There was this sense of here’s this daughter who’s now living with mom and he feels like mom’s pitting her against him and he wants to build a connection (Case 2B).

In these discussions, ambivalence did not necessarily reflect conflicting realities or cognitive dissonance, but in fact may have offered a truer understanding of the situation than attempting to tidily classify the involved parties:

“Love covers a multitude of sins” (1 Peter, 4:8). Your love for your daughter almost covered how much you were just trying to hold it together for yourself, and your love for your mom was almost covering what you were going through (Case 4).

You can traumatize your kids, even if you love them (Case 2B).

The ambivalence apparent in the parent-child dyads and among the participants suggested that ambivalence may be a common dimension of dyadic relationships. In relationships that involve a power differential, whether it is parent-child or doctor-patient, one person depends on the other and the other is depended upon, and such a dynamic seems inclined toward mixed feelings. If that is indeed the case, then perhaps one needs some amount of ambivalence to see the full picture of the other, the good and bad.

Through examining and accepting their own feelings of ambivalence and uncertainty, participants appeared better poised to understand such feelings as experienced by patients. In case 3, which centered on a transitional-age inpatient with type 1 diabetes being seen by the consult-liaison psychiatry team due to concerns about surreptitious insulin use, participants acknowledged and accepted the uncertainty inherent to clinical work in CAP:

Because I worked a lot with adolescents, it was so important for them just to know that you are scared and that it kind of helped them in some way, just to know that someone was being scared for them in between two meetings (Case 3).

The scenario in the following CCPS session (Case 4) centered on a mother who was convinced that her 15-year-old daughter had been abused by her father, despite the daughter’s insistence that her father has not harmed her. Over the course of the simulation, it became apparent that it was less a case about trying to figure out whether or not the abuse had occurred at some point in time:

We’re not lawyers, we’re not court people, we’re not the police. The historical facts aren’t necessarily the most important thing for us and our purposes; it’s trying to figure out what’s going on between the people now, the relationships, and also assessing the safety (Case 4).

In making peace with these feelings of ambivalence and ambiguity, participants invoked the imperative of clinicians to explore and be curious about a patient’s way of understanding the world, their motivations, and their unmet needs, even if it is deeply troubling:

It’s hard for us to be curious about it, and curiosity is important to be therapeutic. Why is he being racist as a defense? Is it not a defense? And I think his story would’ve fleshed that out (Case 5).

Curiosity offered a way forward when participants came up against the limits of their knowledge and perspective, allowing them to accept those limits:

He could have said, ‘You know, you don’t know anything.’ And I think that, yes, guilty as charged. I, of course, cannot understand. But allow me to be interested. Allow me to be interested in you. And maybe it will allow me to help (Case 6).

Curiosity appeared to function as one antidote to the gap between patient and provider as well as the urge to take sides in a parent-child conflict, allowing for a therapeutic relationship with both parties. Ambivalence appeared to be a necessary precursor to curiosity in this context: regardless of one’s initial feelings about a parent or child, positive or negative, reaching a truth that was meaningful to all involved required accepting some ambivalence and ambiguity, that things may not be more complex than they appeared and that apparently conflicting realities could coexist.

### Longitudinal narratives and ambivalence over time

Through the implementation of simulation narratives that carried over across multiple sessions, participants experienced the same simulated family system at two different timepoints in their narrative. We were thus able to examine how participant reflections differed between sessions with the same family and how participants reflected upon the shifting dynamics of the family system. One narrative that was carried over between two sessions (Cases 2 A and 2B, June 2021 and January 2022) involved an adolescent patient who had recently been hospitalized for a suicide attempt and had not been in contact with her father for a few months in the context of her parents’ complicated divorce. In both sessions, participants noted difficulties around helping the daughter be heard due to the charismatic and at times domineering persona of the father:

The dad was hammering at his point and made it difficult sometimes to redirect. That’s why I stepped in and said, “Let’s wait, let [the child] finish,” because she expressed the fear that dad was going to take over. So at that moment, I said I have to be the referee here, and I have to make sure everybody gets their chance to speak (Case 2B).

Additionally, participants between the two sessions had different views on re-establishing the relationship between the estranged father and daughter and what such a reunion would mean for each:

She said, “He’s my dad, of course I want him in my life.“ That was an inflection point where I thought, “Got you,“ because that’s what I wanted to get to, they’re both here, it’s clear they want each other in their lives (Case 2B).

I also wonder what kid doesn’t want a relationship in theory with their parents? It doesn’t mean it’s necessarily going to be helpful. I think every kid desires a relationship with their parent, even if they were being abused by their parent (Case 2 A).

In a similar vein, the issue of power in participants differed in their approach to power breakdown, in this case between the parent and child. Whereas in one session, many participants cast the daughter as a victim, a participant in another session subverted the idea of what constitutes power in this context:

My parents were divorced when I was in middle school. There’s this weird thing when you’re a teenager and you have divorced parents where you actually have a lot of power in terms of where you decide to go. Normally, kids, when they’re teenagers, can’t just ghost a parent for six months. I think the kids are given that choice, and we, as clinicians, often support it (Case 2 A).

For the other longitudinal case, the initial session was in April 2021 with follow-up sessions in November 2021 and June 2022 (Cases 1 A, 1B, and 1 C). This narrative focused on an adolescent experiencing challenges at home and at school in the context of his mother’s recent gender transition. All three sessions depicted the same family system with the same actors at two different timepoints, with Case 1B also including the mother’s new partner, as described above.

Between Cases 1B and 1 C, participants noted the changes in the children and their parents between sessions and how it a created a more dynamic simulated family system:

We don’t know what’s going on exactly, but things just don’t feel right. Whereas last time we could easily pin like okay these two are yelling at each other, everybody’s yelling at each other. And this was just walking into a room and you feel the depression without exactly knowing why the depression (Case 1 C).

Between Cases 1 A and 1 C, interviewers and participants in each session used a similar schema (a child experiencing bullying due to a parent’s minority status) to understand the conflict. However, the emotional meaning and valence given to these interpretations were very distinct and at least in part refracted through personal experiences. During Case 1 A, one observing participant framed the situation in terms of bullying to make sense of it:

He could have had a low-income parent or a parent who is unemployed, or any other situation where kids bully others, because the parents are a minority. He’s dealing with the difficulties of kids who have parents from a minority group (Case 1 A).

Notably, in Case 1B, which continued this patient narrative, bullying was not a point of emphasis. This session involved the patient, his mother, and her new female partner. The dynamics between the male adolescent patient and the two adult women seemed to cause interviewers and observers to be less likely to frame the patient as a victim. In the first and third sessions of this narrative, which included only the patient and his mother, it became evident relatively quickly that the patient’s fights at school in response to peers making negative comments about his mother being transgender. In the first and third sessions, while recognizing the behavior as inappropriate, participants felt that it was rooted in a desire to defend his mother and who she is. However, in the second session, when this aspect of the fights at school came to light, it was framed as bad behavior and expression of the patient’s anger toward his mother and not as him being “clearly protective of his mom,” as one participant put in the first session (Case 1 A). In the third session, a synthesis of the two views emerged, recognizing that both realities could be simultaneously true:

Participant 1: [The patient] might be like, “Mom has nothing to do with this. This is the kids at school.” And so we’re trying to treat two different things as the same thing there. So that makes it even more complicated.

Participant 2: Or mom does have to do with it. But it’s just that she’s causing the bullying. It’s not her fault. He doesn’t necessarily blame her for it. But he probably still feels some resentment towards her for it (Case 1 C).

The longitudinal nature of this case brought to the surface the ambivalence surrounding the patient’s experience of his mother’s transition. Here, the issue of bullying was presented in turns as a way to provide a framework for understanding the patient’s experience and then as a potentially insurmountable challenge. It seemed that both were simultaneously true; as a clinician, one could not necessarily stop the bullying from occurring. But one can address and acknowledge it in the moment and give a name to that experience:

It also brought up a lot of personal memories of being bullied when people found out my dad was a bus driver in middle school. No one could give me any advice about what to do about that because my dad’s not going to stop being a bus driver (Case 1 C).

Often you can’t stop the bad things that happen in the world as a therapist. But I think our job is to help people cope. It’s not your fault. Or exploring why you would think it’s your fault. And some patients need to hear that or be able to have that space (Case 1 C).

Longitudinal narratives in these CCPS sessions provided the opportunity to revisit the patients and their families and to see them with fresh eyes and hear their stories anew. With each new episode of the family narrative, themes recurred but took different forms in different contexts. These multiple valences of meaning became apparent over time in a manner that would be challenging to convey in a single installment of the narrative.

## Discussion

In this qualitative study, we explored the reflections generated by CCPS cases designed to simulate the dyadic and longitudinal dimensions of CAP practice. The emotional experience of the simulations, for interviewers and observers alike, allowed for reflection on personal and professional experiences and triggered meaningful insights and connections between participants. Both the use of two actors and longitudinal narratives across multiple sessions appeared to facilitated unique reflective opportunities. We go on to discuss how these simulated cases called for *emotional labor*, particularly in the form of creating *holding environments*; in this way, the simulated encounters and the debriefing sessions became *dialogic* experiences, in which simulated participants and learners, and learners and instructors could co-construct meaning together.

### Acting like you care: performing emotional labor

Through the CCPS format, participants created clinical situations in psychiatric and dyad-oriented care that called for *emotional labor*, rather than what might typically be considered “clinical work,” i.e., diagnosing and treating illness. In *The Managed Heart: Commercialization of Human Feeling* (1983), Hochschild delineates jobs that are characterized by producing emotional labor by the following criteria: [1] They require interpersonal contact with the public; [2] they require that the worker produce an emotional state in another person; and [3] they allow the employer, through training and supervision, to exercise a degree of control over the emotional activities of employees [19]. Participant reflections suggested that the work of child and adolescent psychiatrists involves modulating the feelings of parents and children while at the same time managing their own emotions. Vinson and Underman frame the demand for emotional labor, i.e., clinical empathy, in the patient encounter as a product of the influence of corporatization (empathy can increase patient satisfaction for the benefit of the whole healthcare organization) and consumerism (patients have greater choice and autonomy in the care and can demand more from their providers) on medicine [20]. In their qualitative analysis of interviews and ethnographic observations of medical students and residents from two U.S. medical schools, the authors describe the shift toward a counterintuitive standardization of empathy and emotional labor within academic and institutional medicine. CCPS appeared to resist such standardization; given that trainees’ challenging experiences provided the source material for the scenarios, each case is individualized and particularized, and no two sessions are identical. The CCPS model was standardized and prescriptive only in terms of the *process* surrounding the production of the simulation, but not in terms of the *content* or the trajectory of the simulated encounter. Nevertheless, the reflections and lessons from any particular simulation session tended to recur among participants across several simulation sessions.

In contrast to this organizational framing, Larson and Yao conceptualize clinical empathy “psychologically” as emotional labor to better understand its components [21]. They argue that empathy is more than an attitude, but is a dynamic process involving affective, cognitive, and behavioral components. Similarly, they define emotional labor as “the process of regulating experienced and displayed emotions to present a professionally desired image during interpersonal transactions at work.” According to the authors, emotional labor, like any form of labor, is not necessarily draining or detrimental to the laborer; it has the potential to be fulfilling and rejuvenating, even as it demands attention and effort. The authors also advocate for using methods of acting to frame empathy for medical trainees. They discuss the role of surface acting, in which “individuals mainly engage in overriding automatic expressions that are not desired, fabricating expressions that are desired, and enduring the dissonance of the two,” and deep acting, in which “memory and imagination are used liberally in an effort to renovate the actor’s inner world, and the role each can play in displaying and experiencing empathy with patients. Additionally, the authors argue that such an approach can aid in avoiding burnout because it “enriches their reservoir of human experience and makes it easier for them to develop perspective.”

While such psychologizing of clinical empathy risks shifting the burden of responsibility from the organizational context onto the individual clinician, it may also offer the possibility of emotional agency. In a similar vein, CCPS aims to prepare trainees “for the daily reality of the ways in which challenging cases often force the clinician to confront the gap between their idealized and empathic clinical self and the self that acted the best they could at the time and under pressure” [8].

### Clinical encounters and debriefings as holding environments

Winnicott offered the view that a psychotherapist can offer healing through a *holding environment*: “A correct and well-timed interpretation in an analytic treatment gives a sense of being held physically that is more real…than if a real holding or nursing had taken place. Understanding goes deeper” [22]. The clinical encounter in CAP can potentially function as a space in which the clinician provides a holding environment for the caregiver-child relationship, to help the caregiver hold the child. In our sessions, participants noted the need to provide, as the clinician, this sort of holding environment for the patient and, at times, the parent. Ziegler and Weidner illustrate the need for interventions for parents of children who have experienced violence to help them debrief the violence and hold the child effectively [23]. The authors describe the particular need in times of crisis for a holding environment for children. In order to provide one successfully, the authors argue that “parents should understand the impact of the trauma on themselves as well as their child and be able to see the child’s needs and emotions when they are different from their own.” They explain that parents may feel responsible for the violence and the resulting guilt and shame can interfere with their ability to empathize with the child, which must be addressed in order to fully address the child’s needs. In our sessions, participants noted the importance of creating a holding environment, with safety, security, empathy, and facilitation as described above, in the clinical space for the child, the parent, or both.

In the context of CCPS, the debriefing session might provide a similar sort of holding environment, to allow trainees to learn and explore with some sense of security. In developing an in-depth debriefing session to address students’ emotions and professional identity formation during simulation, Schweller et al. argued that “since we are advocating the necessity of dealing with patients’ emotions in a positive and constructive way, it is mandatory that facilitators do the same with students’ emotions during the debriefing” [24]. They further compared the debriefing session to that of an ideal clinical encounter, in which one fosters an environment that is “free, safe, devoid of judgment, and based on positive reinforcement” so that “the patient feels at ease to share his or her experiences and thoughts, and so that the doctor has the legitimacy and intimacy necessary to make suggestions and comments that make sense to the patient’s life.” In a similar vein, Ribeiro et al explored debriefing about moral dilemmas with medical students and found that students needed non-judgmental validation of their experiences and difficult emotions as legitimate [25]. Supervising physicians could model how to respond to such situations and communicate the insights they developed. In doing so, they could also show how negative experiences and feelings of ambivalence could become meaningful and transformative: “These doubts are essential and constitutive elements of a conscious and informed choice about the future specialty.”

### A dialogic approach to dyads

The metaphor of the *holding environment*, while useful, has its limits. It suggests one entity holding the other, with a unidirectional flow of care, knowledge, and resources from the “holder” to the “held,” from parent to child, instructor to learner, provider to patient. Yet, these relationships are not one-sided: as the holder changes the held, the held changes the holder. Bakhtin described the concept of *dialogue* as “the single adequate form for verbally expressing authentic human life” [26]. It is such a dialogue that co-construction, as embodied in CCPS, strives to generate. Bakhtin’s dialogic stance has been applied to critical pedagogy, with Freire advocating for dialogic education [27]. Boyd and Markarian characterize Freire’s dialogic teacher as providing “supportive and substantive opportunities for engaged talk with content – to explore, challenge, reconsider, and extend ideas in ways that enhance student learning” [28]. Because CCPS facilitators typically do not generate the simulation scenarios themselves, there may be more room for curiosity and ambiguity in the debriefing sessions, as the facilitator does not have an “answer key.” Rather than specific diagnostic or treatment concerns, the focus tends to become exploring and reflecting upon the emotional and interpersonal work that comes with providing care in challenging clinical scenarios [7]. In considering how these tenets are conducive to the co-construction of knowledge and meaning in the instructor-learner dyad, one could see how they might be applied to the dynamics of parent-child and provider-patient dyads.

A constructionist approach to medicine emphasizes shared meanings in its epistemology, “with truth seen as a dialogic transaction between individuals. As such, truth exists in language and bodily action. […] No one person is in control. Instead, social realities emerge contextually over time [29].” By challenging the dyadic relationship as a false dichotomy, we perhaps could then imagine the dyadic relationships discussed above as singular systems, simultaneously interdependent and at odds with one another, unified by difference. The CCPS format lent itself to a kind of curiosity; the interactions in simulated scenarios and the debriefings were composed of and co-constructed from multiple points of view, including the script writers, the facilitators, the patient actors, and the other participants. In this way, the process of narrative co-construction both called for and generated a sense of ambivalence and curiosity in the simulation and debriefing spaces.

### Challenges and limitations

We concede four main shortcomings. First, we included participants from one CAP training program in an urban academic medical center in the northeastern United States; thus, our findings were likely shaped by the particular nosologies and therapies that make up Western academic child and adolescent psychiatry. Second, our sample of CAP senior fellows may not have been representative in terms of demographics of CAP trainees across the U.S. Though the participant sample was racially and ethnically diverse (with white participants being in the minority), the sample had a large majority of male participants, with only two female participants in the cohort. Third, through the format of group debriefing sessions, we may have introduced the opportunity for groupthink or participants responding in socially desirable ways, issues that individual interviews could have prevented. However, given our interest in how participants make meaning via social interactions, a group format was deemed most appropriate. Lasty, we concede that our learning objectives were broad, not always measurable, and at times aspirational in their quantity.

## Conclusions

In spite of these limitations, this qualitative study explored how the CCPS format and the use of underaged actors and multiple actors facilitated reflection and insight among CAP trainees. The emotional experience of the simulations, for interviewers and observers alike, provided an opportunity to reflect on particularly challenging personal and professional experiences and triggered meaningful insights and connections between participants. These simulated cases called for *emotional labor*, particularly in the form of creating *holding environments*; in this way, the simulated encounters and the debriefing sessions became *dialogic* experiences, in which the patient and provider, parent and child, and learner and instructor could co-construct meaning and foster professional development as reflective practitioners.

## Data Availability

All data generated or analyzed during this study are available upon reasonable request.
